# Factors Associated with Large Renal Function Decline in Patients with Chronic Hepatitis C Successfully Treated with Direct-Acting Antiviral Therapy

**DOI:** 10.3390/diagnostics13030473

**Published:** 2023-01-27

**Authors:** Chun-Hsien Chen, Chien-Heng Shen, Kuo-Liang Wei, Huang-Wei Xu, Wei-Ming Chen, Kao-Chi Chang, Yu-Ting Huang, Yung-Yu Hsieh, Sheng-Nan Lu, Chao-Hung Hung, Te-Sheng Chang

**Affiliations:** 1 Division of Gastroenterology and Hepatology, Department of Internal Medicine, Chang Gung Memorial Hospital, Chiayi 613, Taiwan; 2 College of Medicine, Chang Gung University, Taoyuan 33302, Taiwan

**Keywords:** HCV, direct-acting antiviral therapy, sustained virologic response, renal function

## Abstract

The findings regarding changes in renal function in patients with hepatitis C virus (HCV) infection treated with direct-acting antivirals (DAAs) are controversial. This study attempted to identify the factors associated with the large decline in renal function following DAA treatment. This retrospective cohort study included patients treated with DAAs at Chiayi and Yunlin Chang Gung Hospitals, Taiwan, from 1 January 2017 to 31 October 2020. Estimated glomerular filtration rate (eGFR) data were collected within 90 days prior to DAA therapy and 2 years after the confirmation of a sustained virologic response (SVR). We performed multiple logistic regression to evaluate the clinical or laboratory parameters associated with a large eGFR decline (≥10%). Among the enrolled 606 patients, the mean eGFR at the baseline and endpoint were 84.11 ± 24.38 and 78.88 ± 26.30 mL/min/1.73 m^2^, respectively (*p* < 0.001). The factors associated with a large eGFR decline 2 years after the SVR included hypertension (OR: 1.481; 95% CI: 1.010–2.173, *p* = 0.044) and a higher baseline eGFR (OR: 1.016; 95% CI: 1.007–1.024, *p* < 0.001). A higher albumin level reduced the risk of a large eGFR decline (OR: 0.546; 95% CI: 0.342–0.872, *p* = 0.011). In the patients with HCV treated with DAAs, a larger renal function decline was more commonly observed in those with hypertension, a lower (but within normal range) albumin level, and a higher baseline eGFR, while DAA treatment had no effect. The clinical significance of these findings has to be further defined. Although some risk factors associated with chronic kidney disease may be alleviated after DAA treatment, the regular control and follow-up of risk factors and renal function are still recommended in at-risk patients after HCV eradication.

## 1. Introduction

Hepatitis C virus (HCV) infection is a crucial public health problem globally [1]. In addition to hepatitis B virus (HBV) infection, nonalcoholic fatty liver disease, and alcoholic liver disease, HCV is the main cause of chronic liver disease and liver cancer. HCV infection not only causes liver diseases, such as liver fibrosis, cirrhosis, liver failure, and liver cancer, but also interacts with other risk factors for hepatitis. Moreover, HCV infection worsens the severity of liver disease and accelerates the progression of liver disease. In addition, HCV interferes with the host’s metabolism, including blood sugar regulation and lipid metabolism, thus increasing the risks for many chronic metabolic diseases, such as diabetes mellitus (DM), chronic kidney disease (CKD), cardiovascular disease, and stroke. The successful treatment of HCV infection can reduce the risks of these diseases [2,3,4,5].

In recent years, direct-acting antiviral (DAA) has replaced interferon as the main treatment for HCV. It has the advantages of no need for injection, fewer side effects, a short course of treatment, and a high cure rate (>98%). The use of DAA therapy can significantly reduce the incidence of HCV infection and its related complications [6,7,8].

A concern regarding the use of DAA therapy is its impact on renal function. Studies have reported that the use of DAA therapy to treat HCV infection can prevent or ameliorate renal impairment. In a cohort of 45,260 male patients, the risk of glomerulonephritis was significantly decreased after successful DAA treatment [9]. Another study reported that the annual rate of decline in renal function was lower in patients with HCV and CKD who received DAA treatment [10]. A systematic review indicated that a sustained virologic response (SVR) reduces the risk of kidney disease [11]. However, recent studies have demonstrated that some patients experience worsening renal function during or shortly after DAA therapy [12,13]. After DAA treatment, patients may experience a transient decline in kidney function that improves after treatment ends; however, kidney function declines again after a year of follow-up [14]. Another study including 425 patients divided the trajectories of renal function changes after DAA therapy into six categories. One of these categories including 27% of the cohort exhibited a decline in renal function at the 2-year follow-up; however, no changes in renal function were noted over time in the patients included in the other five categories [15]. Taken together, the findings indicate that not all patients with HCV will experience an improvement in renal function after DAA therapy, and some patients may exhibit completely different changes.

Because of the inconsistency in the findings of previous studies, this study determined the relevant clinical characteristics of patients with worsening renal function after DAA therapy by performing a long-term follow-up and identified the possible predictors of a decline in renal function.

## 2. Materials and Methods

### 2.1. Study Design and Patient Population

**Figure 1:**
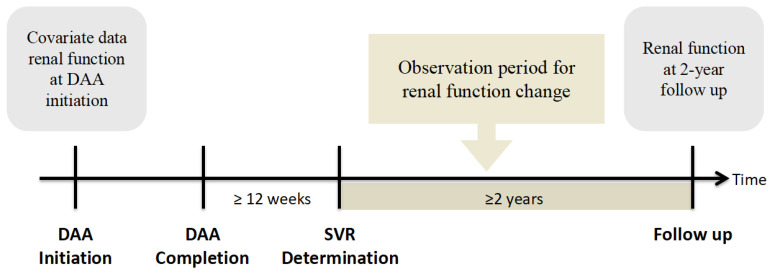
Study design. Abbreviations: DAA, direct-acting antiviral; SVR, sustained virologic response.

In Taiwan, patients with HCV infection are treated with DAAs through a nationwide government-funded program initiated in 2017. The approved DAAs in Taiwan include daclatasvir/asunaprevir with or without ribavirin, ombitasvir/paritaprevir/ritonavir/dasabuvir with or without ribavirin, elbasvir/grazoprevir (Zepatier), glecaprevir/pibrentasvir (Maviret), and sofosbuvir-based (SOF-based) regimens including sofosbuvir + ribavirin, sofosbuvir/ledipasvir (Harvoni), and sofosbuvir/velpatasvir (Epclusa). In this retrospective cohort study, we included patients with HCV who were treated with DAAs at Chiayi and Yunlin Chang Gung Hospitals from 1 January 2017 to 31 October 2020. Patients with a confirmed SVR and available renal function data at the start of DAA therapy and 2 years after SVR determination were included in this study. An SVR was defined as having an HCV RNA level below the lower limit of quantification (LLOQ) at least 12 weeks after the end of DAA therapy (Figure 1). The exclusion criteria were a withdrawal from DAA therapy, a failure to achieve an SVR upon the completion of DAA therapy, the development of end-stage renal disease (ESRD) under maintenance dialysis, death during the study’s follow-up, and the unavailability of renal function data at the endpoint. This study was approved by the Research Ethics Committee of Chang Gung Memorial Hospital and was conducted in accordance with the principles of the Declaration of Helsinki and the International Conference on Harmonization for Good Clinical Practice guidelines.

### 2.2. Assessments and Measurement of Clinical Parameters

From electronic medical records, we collected eligible patients’ history (including medical history and smoking status), physical examination details (including height, weight, and body mass index (BMI)), and laboratory test findings (liver and renal function profiles; hemogram; fasting plasma glucose, albumin; and alpha-fetoprotein (AFP) levels) at baseline. In addition, we obtained details regarding DAA therapy, namely, HCV RNA viral load, genotyping findings, and DAA regimens. The degree of liver fibrosis was assessed by calculating the Fibrosis-4 (FIB-4) index as follows: FIB-4 = [age (years) × AST (UI/L)]/[platelet count (10^9^/L) × ALT (UI/L)^1/2^]. In patients with HCV, we applied a cutoff of <1.45 for the absence of fibrosis or mild fibrosis and one of >3.25 for advanced fibrosis or cirrhosis [16]. The estimated glomerular filtration rate (eGFR) was calculated using the modification of diet in the renal disease equation.

Renal function was examined at two time points: the start of DAA therapy (as the baseline) and 2 years after SVR determination (as the endpoint). Baseline data were defined as the closest measurement within 90 days prior to DAA therapy. Endpoint data were defined as the closest measurement within 90 days after the end of the follow-up. If data were not available, the two closest values within 180 days were interpolated.

A change in renal function was defined as the difference between the eGFR at the baseline and endpoint, whereas the percentage of changes in the eGFR was calculated as the difference divided by the baseline to present the amplitude of the change in renal function. Considering an expected annual decline of 2% to 3% in the eGFR after the SVR on the basis of the findings of previous studies [10] and for the ease of clinical practice, we defined a large eGFR decline as a decrease of ≥10% from baseline to 2 years after SVR determination. No or mild eGFR decline was defined as a decrease of <10% in the eGFR. According to the Kidney Disease Improving Global Outcomes guidelines, the eGFR was classified into five categories to assess the severity of CKD: G1 (≥90 mL/min/1.73 m^2^), G2 (60–89 mL/min/1.73 m^2^), G3 (30–59 mL/min/1.73 m^2^), G4 (15–29 mL/min/1.73 m^2^), and G5 (<15 mL/min/1.73 m^2^) [17].

### 2.3. Statistical Analysis

Continuous data are expressed as the mean (standard deviation), and categorical data are expressed as the number (percentage). The chi-square test and Student’s *t* test were used to compare categorical and continuous parameters among different subgroups, respectively. The paired *t* test was performed to compare renal function and body weight between baseline and endpoints. Univariate and stepwise multivariate logistic regression analyses were performed to evaluate factors related to renal function and body weight changes at endpoints. The results are presented as ORs with 95% CIs. All statistical tests were two tailed, and a *p* value of <0.05 was considered statistically significant. All statistical analyses were performed using SPSS (version 22, IBM, Chicago, IL, USA).

## 3. Results

### 3.1. Baseline Characteristics of the Study Cohort

**Figure 2:**
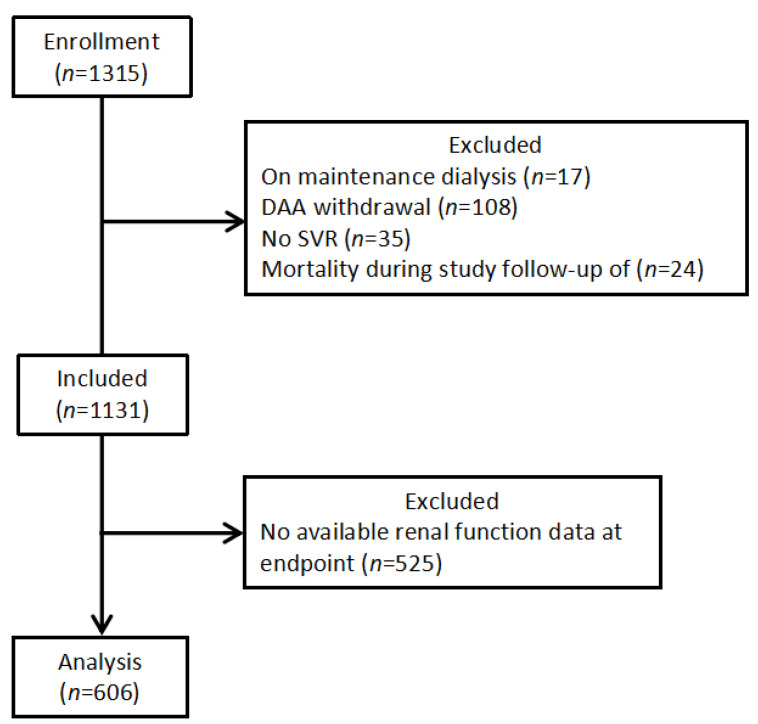
Patient selection flow diagram. Abbreviations: DAA, direct-acting antiviral; SVR, sustained virologic response.

A total of 606 patients were enrolled in this study (Figure 2). Table 1 presents the baseline characteristics of the study population. The mean age of the patients was 67.0 years, 47.9% of the patients were men, the mean HCV-RNA titer was 12.0 × 10^6^ IU/mL, and 436 (71.9%) patients were infected with HCV genotype 1b.

A higher proportion of the patients with a large eGFR decline (a decline of ≥10% from baseline) had hypertension (58.2% vs. 49.0%, *p* = 0.026), a lower albumin level (4.2 vs. 4.3 g/dL, *p* = 0.001), a lower creatinine level (0.87 vs. 0.94 mg/dL, *p* = 0.016), a higher eGFR (87.45 vs. 81.74 mL/min/1.73 m^2^, *p* = 0.005), a higher G1 ratio (42.6% vs. 32.1%, *p* = 0.008), and a lower G2 ratio (44.2% vs. 52.4%, *p* = 0.047) than the patients with no or mild eGFR decline (a decline of <10% from baseline) did.

Table S1 presents the difference between the study cohort and the missing group who had completed DAA treatment with SVR but had no available renal function data at the endpoint (*n* = 525). A higher proportion of the patients in the study group had a younger age (64.9 vs. 67.0 years, *p* = 0.004), a lower smoking ratio (16.8% vs. 22.3%, *p* = 0.021), diabetes mellitus (30.5% vs. 18.9%, *p* < 0.001), hypertension (52.8% vs. 38.9%, *p* < 0.001), dyslipidemia (21.9% vs. 12.4%, *p* < 0.001), hepatocellular carcinoma (10.6% vs. 4.8%, *p* < 0.001), prior interferon use (26.4% vs. 15.4%, *p* = 0.001), a lower albumin level (4.2 vs. 4.3 g/dL, *p* = 0.004), a higher total bilirubin level (0.8 vs. 0.7 mg/dL, *p* < 0.001), a lower platelet count (167,300 vs. 180,200/μL, *p* = 0.001), a higher FIB-4 score (3.67 vs. 3.22, *p* = 0.009), a higher genotype 1b ratio (71.9 vs. 66.1%, *p* = 0.033), a lower genotype 2 ratio (23.1 vs. 30.5%, *p* = 0.005), a higher DCV/ASV ratio (6.3 vs. 2.1%, *p* = 0.001), a higher ProD ratio (19.6 vs. 13.9%, *p* = 0.010), and a lower Maviret ratio (7.3 vs. 18.3%, *p* < 0.001) than the patients in the missing group did.

### 3.2. Renal Function Change

The mean eGFR was 84.11 ± 24.38 mL/min/1.73 m^2^ at baseline and 78.88 ± 26.30 mL/min/1.73 m^2^ at the endpoint (*p* < 0.001).

**Figure 3:**
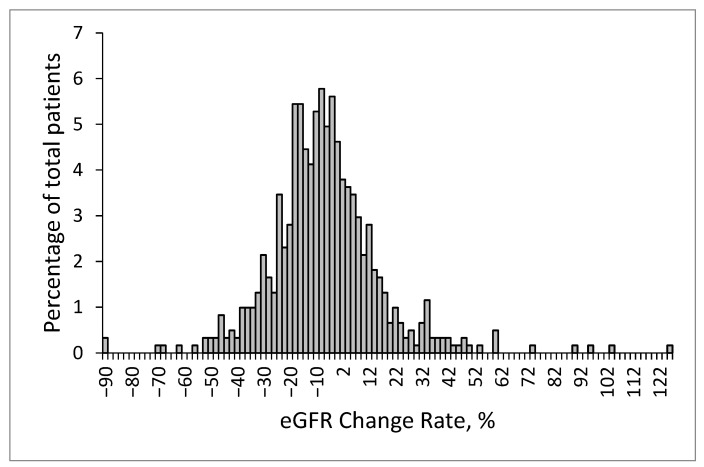
Distribution of changes in the eGFR at the endpoint.

The distribution of changes in the eGFR is presented in Figure 3. The mean change in the eGFR was −5.6% ± 21.2%. The mean change in the eGFR was 6.5% ± 17.4% in the patients with no or mild eGFR decline and −22.8% ± 12.2% in the patients with a large eGFR decline. In the subgroup of the patients with a large eGFR decline, the average eGRF decline rate was −39.8% ± 13.0%.

**Figure 4:**
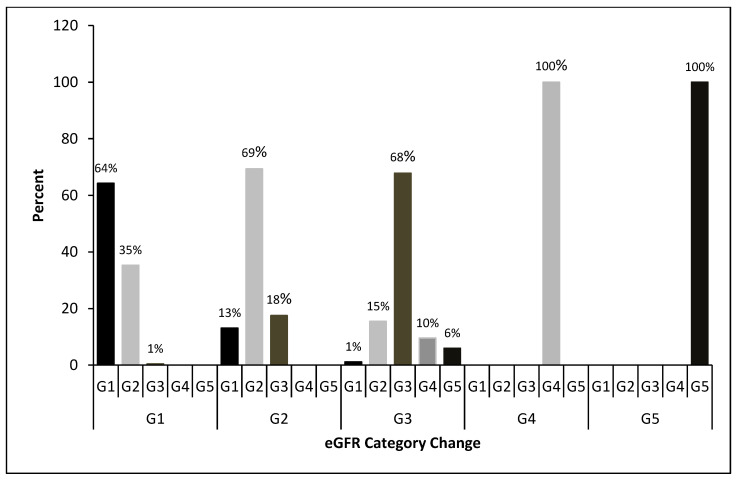
eGFR category changes at the endpoint, by the eGFR category at baseline.

The changes in eGFR categories after the follow-up are presented in Figure 4. Among the 606 patients, 409 (67.4%) exhibited no change in the eGFR category and 144 (23.7%) progressed to a higher eGFR category. Among those with G1 at baseline, 35% were identified as G2 at the endpoint. Among those with G2 at baseline, 18% were identified as G3 at the endpoint. At the endpoint, the proportion of the patients in G2 (15%) was higher than that of the patients in G4 (10%). In the G3 group, 15% improved to G2, 1% improved to G1, 10% deteriorated to G4, and 6% deteriorated to G5.

### 3.3. Multivariable Models for a Large eGFR Decline

The predictors of a large eGFR decline are listed in Table 2. The findings of the univariate analysis indicated that hypertension (*p* = 0.026), a lower albumin level (*p* = 0.002), a higher AFP level (*p* = 0.035), a lower creatinine level (*p* = 0.018), and a higher baseline eGFR (*p* = 0.005) were associated with a large eGFR decline. Compared with G1, G2 had a significantly lower risk of a large eGFR decline (OR: 0.636; 95% CI: 0.447–0.905, *p* = 0.012). The results of the multivariate analysis indicated that hypertension (OR: 1.481; 95% CI: 1.010–2.173, *p* = 0.044) and a higher baseline eGFR (OR: 1.016; 95% CI: 1.007–1.024, *p* < 0.001) were associated with a large eGFR decline 2 years after SVR. By contrast, a higher albumin level reduced the risk of a large eGFR decline (OR: 0.546; 95% CI: 0.342–0.872, *p* = 0.011).

### 3.4. Impact of SOF-Based Treatment on Renal Function

**Figure 5:**
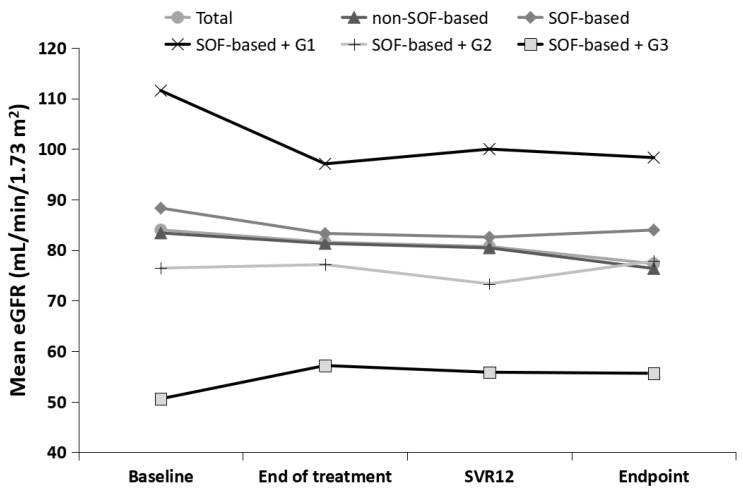
The trend of renal function from baseline to endpoint, stratified by SOF-based or non-SOF-based treatments, and different baseline eGFR categories in SOF-based treatments.

There were 439 patients with complete renal function data at baseline, end of treatment (EOT), 12 weeks after treatment (SVR12), and endpoint. The evolution of renal function from baseline to endpoint is presented in Figure 5 and Table 3.

Regardless of the SOF-based or non-SOF-based treatments, renal function showed a slow decline. In particular, SOF-based treatments had a slightly greater decline in renal function from baseline to EOT, but they were relatively flat from the EOT to the endpoint, whereas non-SOF-based treatments showed a slow decline throughout the follow-up. The mean changes in eGFR from baseline to EOT and from baseline to endpoint were −3.09% and −3.83% in patients with Sof-based treatment, and they were −1.49% and −5.86% in patients with non-Sof-based treatment, respectively.

Regarding the different baseline eGFR categories in SOF-based treatment, renal function continued to decline in G1, remained stable in G2, and initially improved (from baseline to ETR) and then stabilized (from ETR to endpoint) in G3. Contrary to G1, there was no significant difference in renal function at each time point of G2 and G3 compared with the baseline.

## 4. Discussion

In our study, the patients with HCV infection who received DAAs and achieved an SVR had significantly decreased renal function 2 years after the completion of treatment. The annual eGFR decline was −2.61 mL/min/1.73 m^2^, and the decline rate was −2.8%. This value is slightly higher than that reported in a previous study, in which the annual eGFR decline was approximately −2.33 mL/min/1.73 m^2^ in the treated patients with HCV with CKD stage 1 and 2 and −1.32 mL/min/1.73 m^2^ in the patients with CKD stages 3 to 5 [10]. This finding can be attributed to the presence of a greater number of older patients in our cohort. The mean age of the patients in our cohort was 67.0 years compared with 51.9 years in that study. The results of the logistic regression analysis indicated that hypertension, a lower (but within normal range) albumin level, and a higher baseline eGFR independently predicted the risk of a large eGFR decline, whereas the baseline virologic profile and different treatment regimens had no effect.

HCV causes renal damage through multiple mechanisms, including cryoglobulin deposition, immune complex deposition, direct cytopathic effects, increased Toll-like receptor 3 expression in mesangial cells, and increased insulin resistance leading to excess insulin-like growth factor-1 production and mesangial cell proliferation [18]. After HCV eradication, the effects of HCV-related injury diminished or disappeared and were not the primary cause of the large renal function decline observed in our study.

Sofosbuvir, one of the most crucial components of several DAA regimens, is eliminated renally and accumulates in patients with severe CKD or ESRD. Therefore, patients with severe CKD or ESRD were excluded from preapproval clinical trials. However, some studies have reported that patients with CKD did not experience a reduction in the eGFR during or after SOF-based treatment [19,20]. In our study, we found that patients with SOF-based treatment had somewhat more of a renal function decline during the course of treatment than patients with non-SOF-based treatment. The minor trend of eGFR decline during SOF-based treatment was consistent with previous studies [21,22]. However, we found that during the following tracking period, the renal function remained stable in the SOF-based group, while it continued on a slow downward trend after non-SOF-based treatment. Thus, the overall eGFR decline rate was similar in both groups at endpoints. Large cohort studies have demonstrated that patients with preserved renal function during DAA treatment exhibited a significant eGFR decline, whereas patients with advanced CKD experienced a slight improvement in the eGFR [23,24,25,26]. In our study, an improvement in renal function from baseline to EOT was observed in patients receiving SOF-based treatment, with an initial eGFR category of G3. This means that SOF-based treatment did not impair renal function in this group; on the contrary, patients benefited from the treatment, and renal function improved after HCV eradication. Overall, SOF-based treatment did not increase the risk of a substantial decline in renal function over a 2-year follow-up. Our finding is consistent with that of a previous study including 1390 patients and a follow-up period of 2 years; that study observed no effect of DAA treatment on renal function regardless of baseline renal status [27].

The pathophysiology of hypertension in CKD is complex and multifactorial, including volume overload, sympathetic hyperactivity, salt retention, endothelial dysfunction, and alterations in the hormonal system that regulates blood pressure. Long-term uncontrolled hypertension increases the risks of CKD progression and ESRD [28]. HCV is involved in the pathogenesis of atherosclerosis, thereby increasing the incidence of cardiovascular diseases, including coronary artery disease, carotid atherosclerosis, and cerebrovascular disease. The risks of these events are decreased following HCV eradication [29]. Our study revealed that hypertension continued to play a crucial role in CKD after HCV eradication.

HCV may interfere with glucose metabolism and increase the risk of insulin resistance and type 2 DM. Insulin resistance exists in about 80% of patients with HCV infection. When comparing HCV-infected and non-infected patients, HCV-infected patients have twice the risk of developing type 2 DM compared to HCV-uninfected patients [30]. After HCV eradication, insulin resistance and cardiovascular risk are reduced, potentially improving the microvascular complications of DM, including nephropathy [31,32]. In our study, DM was not associated with a large renal function decline at the endpoint. This may imply that insulin resistance and sugar control became better after HCV eradication, which was suggested in previous studies. However, DM alone is a leading cause of CKD progression and ESRD; regular blood sugar monitoring and control is still important after HCV eradication [33].

Our study showed that low (but still within the normal range) albumin at baseline was associated with a higher risk of a decline in renal function after DAA therapies. A slight decline in the albumin level might reflect some condition, including the presence of decreased liver synthesis, chronic inflammation, and malnutrition. Albumin has many physiological functions, including the maintenance of osmotic pressure, the buffering of the acid–base balance, the binding and transport of various compounds, elastase activity, and antioxidant activity [34]. Oxidative stress is one of the main factors causing renal injury in CKD, and a decrease in the albumin value will lead to a decrease in the ability to resist oxidative stress, which will lead to the deterioration of renal function [35]. Most previous studies considered abnormally low albumin levels, namely, hypoalbuminemia, as an indicator of poorer outcomes, including more rapid CKD progression and cardiac events in ESRD [36,37]. One recent large cohort study had the same findings as ours. The study, including 11,000 general population participants, showed that even within the normal range, decreased albumin levels were an independent risk factor for a rapid decline in kidney function [38]. There is currently no specific treatment for decreased albumin values other than treating the underlying disease.

In our study, patients with a higher baseline eGFR exhibited a faster decline in renal function. This finding is similar to that of the Japanese study. In a healthy cohort, patients of different ages but with the same initial renal function had the same rate of decline, whereas, among patients of the same age, those with higher initial renal function had a faster rate of decline. This finding indicates that the rate of decline in renal function is associated with the initial renal function but not age. In severe CKD, the kidneys attempt to maintain physiological function, which may explain this phenomenon [39]. In our study, we found that patients with poor baseline renal function had less of a decline in renal function after 2 years of follow-up. This result was consistent with a 1-year follow-up study. The study included 594 patients treated with sofosbuvir/velpatasvir, and it reported that the persistent deterioration of renal function occurred mainly in patients with diabetes, a baseline eGFR ≥ 60 mL/min/1.73 m^2^, and a concomitant use of RBV [40]. However, the results of two other studies were contrary to ours. A 1-year follow-up study including 1536 patients indicated that liver transplantation and a baseline eGFR < 60 mL/min/1.73 m^2^ were associated with a persistent deterioration in renal function during the study period [41]. Another 24-week follow-up study of 343 patients showed improved renal function at the end of the follow-up, with approximately a 10% reduction in serum creatinine values. In that study, a baseline eGFR ≤ 60 mL/min/1.73 m^2^ was a negative predictor of improved renal function [42]. The reason is unknown. The different study population composition (for example, age, comorbidity, or the presence of other renal diseases, etc.) and length of follow-up may be the reasons. Time is a very important factor. For example, short-term fluctuations in kidney function do not necessarily indicate a long-term sustained decline in kidney function. In addition, the effects of some comorbidities (such as DM or hypertension) on renal function will continue to accumulate over time.

The rate of decline in renal function in patients with CKD was moderated after DAA treatment, and our study demonstrated that nearly one-third (196/606) of patients had improved renal function at the end of the follow-up. In our study, the major risk factors associated with a large renal function decline were multiple traditional risk factors for CKD but not the DAA treatment itself. Indeed, patients treated with DAAs and achieving SVR have many well-documented long-term benefits, including the regression of fibrosis and cirrhosis, a reduced risk of HCC, and an improved risk and control of diabetes, cryoglobulinemia, cardiovascular events, and possibly overall mortality [29,43,44,45]. That is, some risk factors that may contribute to CKD (such as DM and hypertension) may be alleviated after DAA treatment. What we want to emphasize here is that even if the treatment of HCV is successful and the clinical prognosis of many diseases is improved, we should not ignore these risk factors of CKD. For patients with these risk factors before DAA treatment, the regular control and follow-up of risk factors and renal function are recommended after the completion of HCV eradication.

This study has several limitations. First, this is a retrospective study. Because many patients did not return for follow-up regularly after treatment, their renal function data were not available, thereby reducing the number of patients recruited in this study. From the comparison in Table S1, we can find that patients in the missing group had fewer medical diseases and better laboratory test results. Therefore, it is understandable that such patients are less willing to follow up regularly after HCV eradication. Second, the 2-year observation period for this study may be too short, and changes in renal function may have different trajectories when tracked over longer periods of time. Third, comparative studies should be conducted by including matched cohorts of untreated patients with HCV. However, practical difficulties can be encountered. In addition to the lack of a historical control group with a similar follow-up period, not treating patients with HCV for research purposes would be ethically controversial.

## 5. Conclusions

Our study demonstrated that patients with HCV who received DAA treatment and achieved an SVR exhibited a significant decline in renal function after a 2-year follow-up. A larger decline in eGFR is more common in patients with hypertension, a lower (but within normal range) albumin level, and a higher baseline eGFR, but not DAA treatment. The clinical significance of these findings has to be further defined. Although some risk factors associated with CKD may be alleviated after DAA treatment, the regular control and follow-up of risk factors and renal function are still recommended in at-risk patients after HCV eradication.

## Figures and Tables

**Table 1:** Baseline characteristics.

	Total Patients (*n* = 606)	No or Mild eGFR Decline (<10%)	*n*	Large eGFR Decline (≥10%)	*n*	*p* Value
Age, year	67.0 (10.9)	66.5 (11.2)	355	67.8 (10.4)	251	0.165
Male gender, n (%)	290 (47.9%)	180 (50.7%)	355	110 (43.8%)	251	0.095
Body weight, kg	64.3 (12.0)	64.4 (12.2)	346	64.2 (11.7)	245	0.830
BMI, kg/m^2^	25.4 (3.9)	25.3 (3.8)	346	25.5 (4.0)	245	0.596
Current smoking, n (%)	102 (16.8%)	61 (17.2%)	355	41 (16.3%)	251	0.783
Diabetes mellitus, n (%)	185 (30.5%)	102 (28.7%)	355	83 (33.1%)	251	0.254
Hypertension, n (%)	320 (52.8%)	174 (49.0%)	355	146 (58.2%)	251	0.026
Dyslipidemia, n (%)	133 (21.9%)	78 (22.0%)	355	55 (21.9%)	251	0.986
HBsAg positive, n (%)	77 (12.7%)	47 (13.2%)	355	30 (12.0%)	251	0.639
HCC, n (%)	64 (10.6%)	36 (10.1%)	355	28 (11.2%)	251	0.689
Prior IFN, n (%)	160 (26.4%)	98 (27.6%)	157	62 (24.7%)	106	0.391
AST, U/L	61.8 (45.6)	60.7 (45.3)	355	63.4 (46.0)	251	0.473
ALT, U/L	76.1 (71.7)	73.6 (64.5)	355	79.6 (80.8)	251	0.312
Albumin, g/dL	4.2 (0.3)	4.3 (0.3)	355	4.2 (0.3)	251	0.001
Albumin ≤ 3.5 g/dL, n (%)	23 (3.8%)	13 (3.7%)	355	10 (4.0%)	251	0.838
Total bilirubin, mg/dL	0.8 (0.4)	0.8 (0.3)	355	0.8 (0.5)	251	0.586
Platelet, 10^3^/μL	167.3 (65.9)	168.3 (66.1)	355	166.0 (65.6)	251	0.669
FIB-4 score	3.67 (2.86)	3.61 (2.87)	355	3.75 (2.85)	251	0.552
FIB-4 score, n (%)			355		251	
<1.45	112 (18.5%)	71 (20.0%)		41 (16.3%)		0.252
1.45–3.25	237 (39.1%)	130 (36.6%)		107 (42.7%)		0.135
>3.25	257 (42.4%)	154 (43.4%)		103 (41.0%)		0.565
AFP, ng/mL	8.7 (27.3)	6.6 (8.8)	355	11.6 (40.9)	251	0.059
HCV-RNA, 10^6^ IU/mL	12.0 (229.5)	18.6 (299.8)	355	2.7 (3.3)	251	0.399
HCV-RNA, log-transformed	5.9 (0.9)	5.9 (0.9)	355	5.9 (1.0)	251	0.418
HCV genotype, n (%)			355		251	
1a	9 (1.5%)	5 (1.4%)		4 1.6(%)		1.000
1b	436 (71.9%)	260 (73.2%)		176 (70.1%)		0.400
2	140 (23.1%)	81 (22.8%)		59 (23.5%)		0.843
3	1 (0.2%)	0 (0%)		1 (0.4%)		0.414
6	8 (1.3%)	4 (1.1%)		4 (1.6%)		0.724
mixed	12 (2.0%)	5 (1.4%)		7 (2.8%)		0.250
DAAs, n (%)			355		251	
DCV/ASV	38 (6.3%)	22 (6.2%)		16 (6.4%)		0.929
ProD	119 (19.6%)	73 (20.6%)		46 (18.3%)		0.495
Zepatier	217 (35.8%)	134 (37.7%)		83 (33.1%)		0.237
Maviret	45 (7.4%)	22 (6.2%)		23 (9.2%)		0.170
SOF-based	187 (30.9%)	104 (29.3%)		83 (33.1%)		0.322
Ribavirin use, n (%)	87 (14.4%)	46 (13.0%)	355	41 (16.3%)	251	0.243
Hemoglobin, g/dL	13.8 (1.7)	13.9 (1.7)	355	13.7 (1.7)	251	0.150
Fasting plasma glucose, mg/dL	115.8 (39.3)	115.3 (37.9)	200	116.6 (41.5)	135	0.768
Creatinine, mg/dL	0.91 (0.33)	0.94 (0.34)	355	0.87 (0.32)	251	0.016
eGFR, mL/min/1.73 m^2^	84.11 (24.38)	81.74 (22.82)	355	87.45 (26.10)	251	0.005
eGFR category, n (%)			355		251	
G1: ≥90 mL/min/1.73 m^2^	221 (36.5%)	114 (32.1%)		107 (42.6%)		0.008
G2: 60–89 mL/min/1.73 m^2^	297 (49.0%)	186 (52.4%)		111 (44.2%)		0.047
G3: 30–59 mL/min/1.73 m^2^	84 (13.9%)	52 (14.6%)		32 (12.7%)		0.505
G4: 15–29 mL/min/1.73 m^2^	3 (0.5%)	3 (0.8%)		0 (0%)		0.271
G5: <15 mL/min/1.73 m^2^	1 (0.2%)	0 (0%)		1 (0.4%)		0.414

Data are expressed as the mean (standard deviation) or number (percentage). Abbreviations: AFP, alpha-fetoprotein; ALT, alanine aminotransferase; AST, aspartate transaminase; BMI, body mass index; DAA, direct-acting antiviral; DCV/ASV, daclatasvir/asunaprevir; eGFR, estimated glomerular filtration rate; FIB-4, fibrosis index based on four factors; HCC, hepatocelluar carcinoma; IFN, interferon; ProD, paritaprevir/ritonavir/ombitasvir/dasabuvir; SOF, sofosbuvir.

**Table 2:** Logistic regression analysis for a large eGFR decline.

Variables	Univariate		Multivariate	
	OR (95%CI)	*p* Value	OR (95%CI)	*p* Value
Age, year	1.011 (0.996–1.026)	0.166	1.015 (0.997–1.034)	0.113
Gender, Male vs. Female	0.758 (0.548–1.049)	0.095	0.770 (0.544–1.090)	0.141
Body weight, kg	0.999 (0.985–1.012)	0.830		
BMI, kg/m^2^	1.011 (0.970–1.054)	0.596	1.016 (0.970–1.064)	0.505
Current smoking, Yes vs. No	0.941 (0.610–1.452)	0.783		
Diabetes mellitus, Yes vs. No	1.225 (0.864–1.738)	0.254	1.283 (0.878–1.876)	0.198
Hypertension, Yes vs. No	1.446 (1.044–2.004)	0.026	1.481 (1.010–2.173)	0.044
Dyslipidemia, Yes vs. No	0.997 (0.674–1.473)	0.986		
HBsAg positive, Yes vs. No	0.890 (0.545–1.451)	0.639		
HCC, Yes vs. No	1.113 (0.660–1.876)	0.689		
AST, U/L	1.001 (0.998–1.005)	0.473		
ALT, U/L	1.001 (0.999–1.003)	0.314		
Albumin, g/dL	0.500 (0.325–0.768)	0.002	0.546 (0.342–0.872)	0.011
Total bilirubin, mg/dL	1.099 (0.781–1.548)	0.588		
Platelet, 10^3^/μL	0.999 (0.997–1.002)	0.668		
FIB-4 score	1.017 (0.962–1.076)	0.552		
<1.45	Reference			
1.45–3.25	1.425 (0.898–2.262)	0.133		
>3.25	1.158 (0.732–1.832)	0.530		
AFP, ng/mL	1.014 (1.001–1.026)	0.035	1.007 (0.995–1.019)	0.258
HCV-RNA, 10^6^ IU/mL	0.999 (0.996–1.002)	0.644		
HCV genotype				
1a	NA ^a^			
1b	Reference			
2	1.076 (0.731–1.583)	0.710		
3	NA ^a^			
6	NA ^a^			
Mixed	NA ^a^			
DAAs				
DCV/ASV	0.911 (0.450–1.845)	0.796		
ProD	0.790 (0.494–1.261)	0.323		
Zepatier	0.776 (0.521–1.155)	0.212		
Maviret	1.310 (0.683–2.514)	0.417		
SOF-based	Reference			
SOF-based, Yes vs. No	1.192 (0.842–1.689)	0.322	1.190 (0.754–1.878)	0.455
Ribavirin use, Yes vs. No	1.311 (0.831–2.069)	0.244	1.123 (0.614–2.054)	0.706
Hemoglobin, g/dL	0.933 (0.849–1.025)	0.151		
Fasting plasma glucose, mg/dL	1.001 (0.995–1.006)	0.767		
Creatinine, mg/dL	0.510 (0.293–0.890)	0.018		
eGFR, mL/min/1.73 m^2^	1.010 (1.003–1.017)	0.005	1.016 (1.007–1.024)	<0.001
eGFR category, n (%)				
G1: ≥90 mL/min/1.73 m^2^	Reference			
G2: 60–89 mL/min/1.73 m^2^	0.636 (0.447–0.905)	0.012		
G3: 30–59 mL/min/1.73 m^2^	0.656 (0.392–1.095)	0.107		
G4: 15–29 mL/min/1.73 m^2^	NA ^a^			
G5: <15 mL/min/1.73 m^2^	NA ^a^			

NA, not applicable. ^a^ Too few cases.

**Table 3:** Renal function at baseline, end of treatment, SVR12, and endpoint.

	Baseline	End of Treatment	SVR12	Endpoint
Total (*n* = 439)	83.99 ± 24.65	81.55 ± 23.42	80.69 ± 24.76	77.30 ± 24.55
	Reference	*p* < 0.001	*p* < 0.001	*p* < 0.001
Non-SOF-based (*n* = 384)	83.37 ± 24.51	81.30 ± 23.94	80.43 ± 24.82	76.35 ± 24.62
	Reference	*p* = 0.001	*p* < 0.001	*p* < 0.001
SOF-based (*n* = 55)	88.29 ± 25.43	83.28 ± 19.54	82.55 ± 24.46	83.96 ± 23.20
	Reference	*p* = 0.037	*p* = 0.007	*p* = 0.073
G1 (*n* = 23)	111.54 ± 19.71	97.05 ± 18.11	99.97 ± 23.91	98.28 ± 21.72
	Reference	*p* = 0.002	*p* = 0.001	*p* < 0.001
G2 (*n* = 26)	76.43 ± 7.89	77.13 ± 12.07	73.32 ± 15.09	77.83 ± 17.58
	Reference	*p* = 0.776	*p* = 0.314	*p* = 0.700
G3 (*n* = 6)	50.58 ± 6.40	57.16 ± 8.04	55.83 ± 10.44	55.62 ± 9.46
	Reference	*p* = 0.137	*p* = 0.317	*p* = 0.248

*p* value is comparing the difference in eGFR between the baseline (reference) and the indicated point.

## Data Availability

The data presented in this study are available on request from the corresponding author.

## Supplementary Materials

The following supporting information can be downloaded at: https://www.mdpi.com/article/10.3390/diagnostics13030473/s1, Table S1: Comparison between study cohort and missing group.
