# Supplementary material for: Factors Associated with Large Renal Function Decline in Patients with Chronic Hepatitis C Successfully Treated with Direct-Acting Antiviral Therapy
Source: Diagnostics (Basel). 2023 Jan 27;13(3):473. doi: 10.3390/diagnostics13030473 (PMC9914858; doi:10.3390/diagnostics13030473)
Supplement: Supplementary file 1 [file diagnostics-13-00473-s001.zip › diagnostics-2152347-supplementary.pdf]

Supplementary Tables

Table S1 Comparison between study cohort and missing group

	**Total patients (*n*=606)**	***n***	**Missing group (*n*=525)**	***n***	***p* Value**
Age, year	67.0 (10.9)	606	64.9 (13.3)	525	0.004
Male gender, n (%)	290 (47.9%)	606	233 (44.4%)	525	0.243
Body weight, kg	64.3 (12.0)	591	63.9 (13.1)	513	0.584
BMI, kg/m^2	25.4 (3.9)	591	25.2 (4.1)	513	0.521
Current smoking, n (%)	102 (16.8%)	606	117 (22.3%)	525	0.021
Diabetes Mellitus, n (%)	185 (30.5%)	606	99 (18.9%)	525	< 0.001
Hypertension, n (%)	320 (52.8%)	606	204 (38.9%)	525	< 0.001
Dyslipidemia, n (%)	133 (21.9%)	606	65 (12.4%)	525	< 0.001
HBsAg positive, n (%)	77 (12.7%)	606	63 (12.0%)	525	0.719
HCC, n (%)	64 (10.6%)	606	25 (4.8%)	525	< 0.001
Prior IFN, n (%)	160 (26.4%)	263	81 (15.4%)	180	0.001
AST, U/L	61.8 (45.6)	606	58.3 (48.2)	525	0.204
ALT, U/L	76.1 (71.7)	606	71.1 (66.2)	525	0.222
Albumin, g/dL	4.2 (0.3)	606	4.3 (0.3)	524	0.004
Albumin ≤ 3.5 g/dL, n (%)	23 (3.8%)	606	17 (3.2%)	524	0.617
Total bilirubin, mg/dL	0.8 (0.4)	606	0.7 (0.3)	525	< 0.001
Platelet, $10^3/\mu L$	167.3 (65.9)	606	180.2 (67.4)	525	0.001
FIB-4 score	3.67 (2.86)	606	3.22 (2.89)	525	0.009

FIB-4 score, n (%)		606		525	
< 1.45	112 (18.3%)		128 (24.4%)		0.016
1.45 - 3.25	237 (39.1%)		208 (39.6%)		0.861
> 3.25	257 (42.4%)		189 (36.0%)		0.028
AFP, ng/mL	8.7 (27.2)	606	7.7 (22.1)	521	0.495
HCV-RNA, 10^6 IU/mL	12.0 (229.5)	606	2.9 (5.1)	525	0.368
HCV-RNA, log-transformed	5.9 (0.9)	606	5.8 (1.0)	525	0.613
HCV genotype, n (%)		606		525	
1a	9 (1.5%)		12 (2.3%)		0.380
1b	436 (71.9%)		347 (66.1%)		0.033
2	140 (23.1%)		160 (30.5%)		0.005
3	1 (0.2%)		3 (0.6%)		0.342
6	8 (1.3%)		16 (3.0%)		0.061
mixed	12 (2.0%)		15 (2.9%)		0.335
DAAs, n (%)		606		525	
DCV/ASV	38 (6.3%)		11 (2.1%)		0.001
ProD	119 (19.6%)		73 (13.9%)		0.010
Zepatier	217 (35.8%)		188 (35.8%)		1.000
Maviret	45 (7.4%)		96 (18.3%)		< 0.001
SOF-based	187 (30.9%)		157 (29.9%)		0.728
Ribavirin use, n (%)	87 (14.4%)	606	56 (10.7%)	525	0.063
Hemoglobin, g/dL	13.8 (1.7)	606	13.8 (1.7)	525	0.874
Fasting plasma glucose, mg/dL	115.8 (39.3)	335	114.6 (40.8)	243	0.720
Creatinine, mg/dL	0.91 (0.33)	606	0.90 (0.40)	525	0.670

eGFR, mL/min/1.73m^2	84.11 (24.38)	606	85.92 (24.19)	525	0.211
eGFR category, n (%)		606		525	
G1: ≥ 90 mL/min/1.73m^2	221 (36.5%)		218 (41.5%)		0.082
G2: 60 - 89 mL/min/1.73m^2	297 (49.0%)		234 (44.6%)		0.136
G3: 30 - 59 mL/min/1.73m^2	84 (13.9%)		65 (12.4%)		0.463
G4: 15 - 29 mL/min/1.73m^2	3 (0.5%)		6 (1.1%)		0.317
G5: < 15 mL/min/1.73m^2	1 (0.2%)		2 (0.4%)		0.600

Data are expressed as the mean (standard deviation) or number (percentage).

Abbreviations: AFP, alpha-fetoprotein; ALT, alanine aminotransferase; AST, aspartate transaminase; BMI, body mass index; DAA, direct-acting antiviral; DCV/ASV, daclatasvir/asunaprevir; eGFR, estimated glomerular filtration rate; FIB-4, fibrosis index based on four factors; HCC, hepatocelluar carcinoma; IFN, interferon; ProD, paritaprevir/ritonavir/ombitasvir/dasabuvir; SOF, sofosbuvir.
